# Diagnostic Accuracy of Nonmydriatic Fundus Photography for the Detection of Glaucoma in Diabetic Patients

**DOI:** 10.1155/2015/892174

**Published:** 2015-10-18

**Authors:** Francisco J. Muñoz-Negrete, Inés Contreras, Noelia Oblanca, M. Dolores Pinazo-Durán, Gema Rebolleda

**Affiliations:** ^1^Hospital Universitario Ramón y Cajal, Instituto Ramón y Cajal de Investigaciones Sanitarias (IRYCIS), 28034 Madrid, Spain; ^2^Universidad de Alcalá, Alcalá de Henares, Spain; ^3^Ocular Pathology National Net (OFTARED) of the Institute of Health Carlos III, Madrid, Spain; ^4^Ophthalmology Research Unit “Santiago Grisolía”, Faculty of Medicine and Odontology, University of Valencia, Valencia, Spain

## Abstract

*Purpose.* To determine the diagnostic accuracy for glaucoma of a set of criteria with nonmydriatic monoscopic fundus photography (NMFP) in diabetics.* Methods.* Diabetics recruited from a screening program for diabetic retinopathy and diabetic glaucoma patients recruited from our glaucoma unit were included. Any patient with evidence of diabetic retinopathy was excluded. Diabetic patients had to have no visual field defects to be included as controls. Glaucoma patients had to have a glaucomatous field defect in at least one eye to be included. One NMFP was taken per eye for all subjects. These photographs were evaluated by two masked glaucoma specialists for the presence of the following: bilateral cup to disc (C/D) ratio ≥0.6, notching or thinning of the neuroretinal rim, disc hemorrhages, and asymmetry in the C/D ratio between both eyes ≥0.2. This evaluation led to a dichotomous classification: if any of the above criteria was present, the patient was classified as glaucoma. If none were present, the patient was classified as normal.* Results.* 72 control subjects and 72 glaucoma patients were included. Evaluation of NMFP had a sensitivity of 79.17% and a specificity of 80.56% for specialist 1 and a sensitivity of 72.22% and a specificity of 88.88% for specialist 2 for the detection of glaucoma. The overall accuracy was 79.83% and 80.55%, respectively.* Discussion.* NMFP evaluation by a glaucoma specialist may be useful for the detection of glaucoma in diabetics.

## 1. Introduction

Open-angle glaucoma is one of the leading causes of irreversible visual loss worldwide [1]. Globally, there are an estimated 60 million people with glaucomatous optic neuropathy and an estimated 8.4 million people who are blind as a result of glaucoma. These numbers are set to increase to 80 million and 11.2 million by 2020 [2]. Because initial glaucoma is asymptomatic, approximately 50% of patients with glaucoma are unaware that they suffer a disease that can lead to blindness if the condition goes untreated [3, 4]. Efforts have been made to develop screening programs for glaucoma; however, there is insufficient economic evidence on which to base recommendations regarding screening for glaucoma [5]. Nevertheless, targeted screening of subgroups at higher risk of developing glaucoma may be viable. One such group may be diabetic patients. Although there is conflicting evidence, a meta-analysis published in 2004 reported that diabetic patients are at significantly increased risk of developing glaucoma [6].

Screening for diabetic retinopathy is now often performed through nonmydriatic fundus photography [7–9]. Although the primary objective is to detect patients with diabetic retinopathy which requires an evaluation by a retina specialist, the graders may also assess the optic nerve. Patient referral in each healthcare system varies, but patients with anomalous optic nerves will probably be referred for further investigation to glaucoma units. A recent publication has evaluated the glaucoma referrals from a local unit of the English National Screening Programme for Diabetic Retinopathy, reporting a positive predictive value of 78.8% at detecting glaucoma of dilated fundus photography [10]. In contrast, in a large survey to determine the outcomes resulting from optometric referrals, only one in five subjects had glaucoma [11]. Thus, Ong et al. suggest that the opportunity of using images taken during diabetic retinopathy screening to detect glaucoma in one of the highest risk target populations should not be missed. However, there remain several obstacles: one of them is that the evaluation of the optic nerve is highly subjective. Another is that the diagnostic accuracy of fundus photography is still unknown.

In an attempt to reduce the subjectiveness of optic nerve assessment, we designed this study, in which a set of criteria for detecting glaucomatous damage was employed. Our aim was to evaluate the accuracy of optic nerve head evaluation using these criteria in nonmydriatic fundus photography (which is the method for diabetic retinopathy screening in our sanitary area) for the diagnosis of glaucoma in diabetic patients.

## 2. Methods

This study was designed as a case-control study; this design was chosen because of the low prevalence of glaucoma even in a high-risk population such as diabetics. Ethics committee approval was obtained; the study adhered to the tenets of the declaration of Helsinki. All patients were informed of the nature of the investigation and signed a written consent form. Diabetic nonglaucomatous subjects, which will be referred to from now on as control subjects, were randomly recruited from diabetic patients sent to our hospital's screening program for diabetic retinopathy. Glaucoma patients with diabetes were recruited from our hospital's glaucoma service.

All study subjects underwent an extensive ophthalmologic evaluation, including nonmydriatic fundus photography, best-corrected visual acuity (BCVA), intraocular pressure (IOP) measurement with a Goldmann applanation tonometer, central corneal thickness measurement with contact ultrasonography, anterior and posterior pole biomicroscopy (with cup-to-disc (C/D) ratio estimation after pupillary dilation), visual field testing, and optical coherence tomography (OCT) scanning of the optic nerve head. Visual fields were performed on a Humphrey perimeter with the Swedish Interactive Threshold Algorithm 24-2 strategy (Carl-Zeiss Meditec, Dublin, California, USA). Visual fields were considered reliable if there were fewer than 20% false-positive responses, false-negative responses, and fixation loses. Optical coherence tomography was performed and analysed with the Optic Disc Cube 200 × 200 protocol of Cirrus OCT (Carl-Zeiss Meditec, Dublin, California, USA). This protocol provides, in addition to other measurements, the average retinal nerve fiber layer (RNFL) thickness, disc area, and vertical C/D ratio.

Fundus photographs were taken by nurses trained by a technician and an ophthalmologist on how to operate a nonmydriatic fundus camera (TRC-NW200, Topcon Europe Medical, Netherlands). Ideally, one image of the posterior pole, including the optic nerve head and the macula, was to be taken for both eyes of all participants. Images were then forwarded through a safe telematic line to a tertiary care hospital, where they were arranged in order to be assessed in alphabetical order with no imposed time limits by two masked glaucoma specialist observers (Gema Rebolleda and Francisco J. Muñoz-Negrete). Another glaucoma specialist (Inés Contreras) oversaw all the examinations performed.

Subjects were classified as controls if no evidence was found of diabetic retinopathy or any other ocular disease apart from mild cataracts. Visual fields had to be reliable, with no glaucomatous defects. No cut-off point was set for C/D ratio in order for a subject to be included as a control. Since this study focused on the ability of fundus photography to detect glaucoma, including control subjects with previously normal C/D ratios was likely to bias the results in favour of subjective assessment of the optic disc. Glaucoma subjects had to have a reproducible glaucomatous visual field defect in at least one eye, defined as two or more contiguous points with a pattern deviation *P* < 0.01 sensitivity loss or more, or three or more contiguous points with *P* < 0.05 sensitivity loss or more, in the superior or inferior arcuate areas or an abnormal result in a glaucoma hemifield test. Glaucomatous field defect severity was classified according to the Hodapp classification [12]. In order to be included in the study, glaucomatous subjects must not have any evidence of any other ocular pathology, apart from mild cataracts. Exclusion criteria for both controls and glaucomatous subjects were the presence of a BCVA of less than 20/40 and of a spherical equivalent of more than 5 diopters.

**Figure 1:**
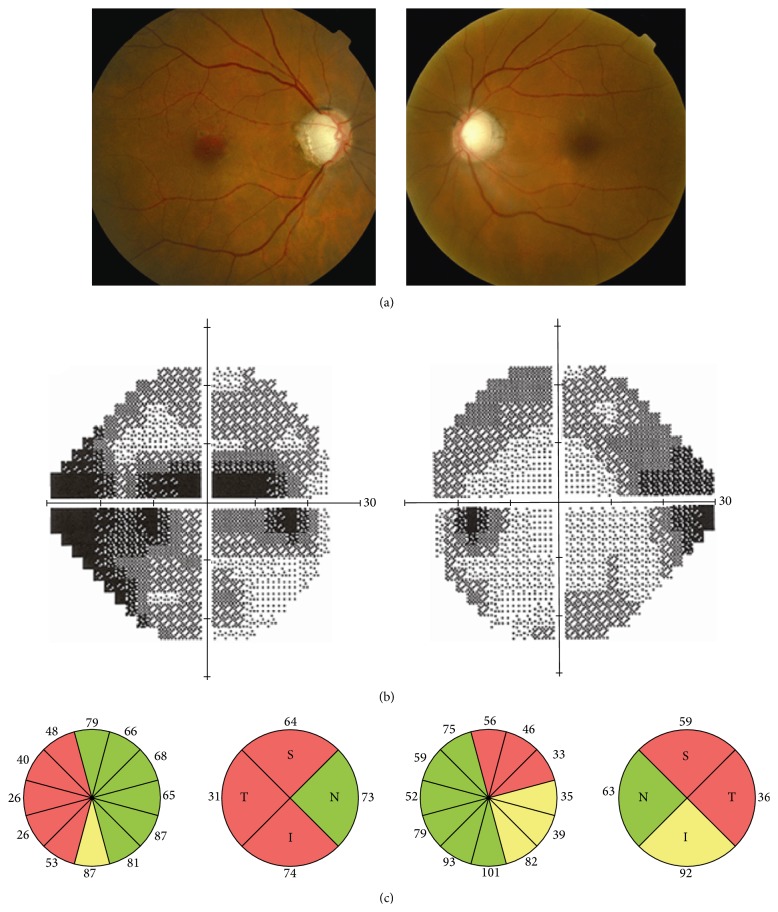
Nonmydriatic fundus photography, visual fields, and optical coherence tomography of a 77-year-old woman. Cup-to-disc ratio is 0.9 in both eyes, visual fields show severe diffuse defects, and optical coherence tomography reflects severe retinal nerve fiber layer loss in both eyes. Intraocular pressure was 23 mmHg in the right eye and 22 mmHg in the left eye. She was diagnosed with bilateral glaucoma.

Fundus photographs obtained by nonmydriatic fundus photography were presented for evaluation to two experienced observers who were masked to participant clinical details and the proportion of control and glaucoma participants. The observers were asked to evaluate each pair of images for the presence of the following criteria of glaucomatous optic nerve damage (adapted from O'Connor et al. [13]):

- bilateral C/D ratio of 0.6 or higher (Figure 1),
- notching or thinning of the neuroretinal rim,
- disc haemorrhages (defined as hemorrhages at the border of the optic, running parallel to the nerve fibers in the nerve fiber layer, shaped liked splinters),
- asymmetry in the C/D ratio between the two eyes of 0.2 or higher.

**Figure 2:**
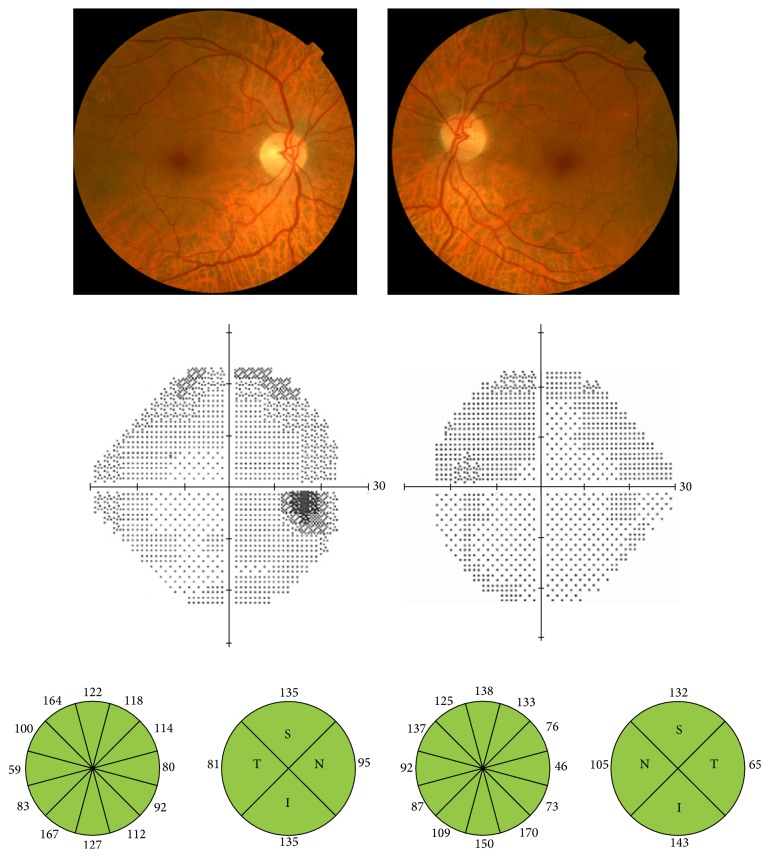
Nonmydriatic fundus photography, visual fields, and optical coherence tomography of a 68-year-old man. Cup-to-disc ratio is 0.1 in both eyes, visual fields are normal, and optical coherence tomography is within normal limits.

This evaluation led to a dichotomous classification: if any of the above criteria was present, the patient was classified as glaucoma (Figure 1). If none were present, the patient was classified as normal (Figure 2). The observers were also asked to estimate the vertical C/D ratio for each eye. In order to estimate intraobserver variability, specialist 2 was asked to reevaluate the photographs of 50 subjects chosen randomly.

### 2.1. Statistics

Sample size calculation: previous reports of stereoscopic evaluation of the optic nerve head by glaucoma specialists have shown that its sensitivity for the diagnosis of glaucoma ranges between 70 and 80% for a specificity of 80% [14–16]. Our working hypothesis was that nonmydriatic fundus photography could reach similar values. Thus, for an expected sensitivity of 70%, a precision of 10%, and a confidence level of 95% the number of glaucoma patients required would be 72 patients. Since the design of the study was a one to one case control, 72 healthy control subjects would be necessary for an expected specificity of 70%.

The sensitivity, specificity, and overall accuracy of the evaluation by the glaucoma specialists of nonmydriatic fundus photography for the diagnosis of glaucoma were calculated. Sensitivity was obtained dividing the number of glaucoma patients with signs of glaucomatous optic nerve damage on nonmydriatic fundus photography by the total number of glaucoma patients. Specificity was obtained by dividing the number of controls who had no signs of optic nerve damage on nonmydriatic fundus photography by the total number of controls. Overall accuracy was defined as the sum of true positives and true negatives divided by the total number of subjects. Cohen's kappa test was calculated to estimate the agreement between the two observers as well as intraobserver variability. The SPSS for Windows software, version 12.0 (SPSS, Chicago, IL), was used to perform the statistical analysis.

## 3. Results

In order to fulfill the sample size requirements, 72 consecutive glaucoma cases and 72 consecutive control subjects were included. All participants were Caucasian. Eighty-six participants were men and 58 women. Among cases there were 40 men (55.6%) and among controls 46 (63.9%), although this difference was not statistically significant (*P* = 0.396, Chi-Square). Control subjects were slightly younger than glaucoma patients (66.1 ± 6.1 years versus 68.3 ± 8.0 years, resp.); the difference was not statistically significant (*P* = 0.065). Glaucoma was unilateral in 17 patients (23.6% of glaucoma subjects). Visual field damage in the most affected eye was mild in 22 (30.6%), moderate in 27 (37.5%), and severe in 23 patients (31.9%). Mean central corneal thickness was similar in cases and controls: 554 *μ*m (SD 36.0) and 556 *μ*m (SD 31.5). However, there was a statistically significant difference between eyes with primary open-angle glaucoma and eyes with normotensive glaucoma: 557 *μ*m (SD 31.7) and 545 *μ*m (SD 42.8), respectively, *P* = 0.021.

For specialist 1, 14 patients (19.44%) of the control group and 57 (79.17%) of the glaucoma group showed at least one of the criteria of optic nerve glaucomatous damage; the figures for specialist 2 were 8 patients (11.11%) in the control group and 52 (72.22%) in the glaucoma group. The frequency with which each of the criteria of optic nerve damage was found is recorded in Table 1, together with their sensitivity and specificity. The most sensitive criterion was the presence of a C/D ratio ≥0.6 for specialist 1 and the presence of cup-to-disc asymmetry for specialist 2 (Table 2). The presence of notches or thinning of the neuroretinal rim and that of optic disc hemorrhages both had a specificity close to 100%. However, these findings were less frequent, especially the presence of hemorrhages, resulting in a very low sensitivity. Evaluation of NMFP with the proposed criteria had a sensitivity of 79.17% (95% confidence interval (95% CI) 69.79%–88.55%) and a specificity of 80.56% (95% CI 71.41%–89.70%) for specialist 1 and a sensitivity of 72.22% (95% CI 61.87%–82.56%) and a specificity of 88.88% (95% CI 81.62%–96.14%) for specialist 2 for the detection of glaucoma. The overall accuracy was 79.83% and 80.55%, respectively. The agreement between both glaucoma specialists was high, with a kappa value of 0.763, *P* < 0.01. Intraobserver variability was also high, with a kappa value of 0.830, *P* < 0.01.

A careful analysis of the misdiagnosis of nonmydriatic fundus photography was performed. Fifteen cases were undetected by specialist 1 (false negatives). In six patients this was due to an underestimation of the C/D ratio by the glaucoma specialist that evaluated the photographs. Four of these pairs of photographs had a very low quality. In the other two pairs, the cup size was difficult to estimate because of diffuse pallor. In the remaining nine patients, the C/D ratio estimated by the glaucoma specialist was similar to that estimated by biomicroscopic evaluation: these were patients in which there was no detectable increase in C/D ratio despite the presence of glaucomatous field defects. Visual field damage in the most affected eye in these “missed” cases was mild in 7, moderate in 3, and severe in 5 patients. Specialist 2 “missed” 20 patients. In 10 cases there was no detectable increase in C/D ratio, and in the other 10 cases the specialist underestimated C/R ratio. Visual field damage in the most affected eye in these “missed” cases was mild in 8, moderate in 6, and severe in 6 patients.

On the other hand, 14 controls were classified as glaucoma by specialist 1 (false positives). Seven subjects had a C/D ratio ≥0.6 and the other 7 had an asymmetry in C/D ratio. Biomicroscopic evaluation by a glaucoma specialist agreed with the C/D evaluation of specialist 1 in 13 subjects; only in one control was the asymmetry in the C/D ratio overestimated. Specialist 2 classified 8 controls as cases: 5 subjects had a C/D ratio ≥0.6 and 2 had an asymmetry in C/D ratio. Again, only in one control was the asymmetry in the C/D ratio overestimated. Mean optic disc size as estimated by OCT in control subjects with a C/D ratio ≥0.6 was 2.96 mm^2^ (SD 0.49; range 2.10–3.64), compared to a mean of 1.94 mm^2^ (SD 0.39) for all other study eyes; that is, these eyes had macrodiscs. The six controls with a real asymmetry in C/D ratio seemed to have a physiologic asymmetry.

## 4. Discussion

Nonmydriatic fundus photography has been proven to be an adequate method for screening for diabetic retinopathy. It makes screening available to more patients at a lower cost than conventional in-office evaluation by an ophthalmologist, and it is more convenient for patients and helps to reduce the burden on ophthalmology services [17]. Glaucoma is a disease that is initially asymptomatic and it is estimated that approximately 50% of patients are unaware that they suffer from this disease [3, 4]. Efforts have been made to develop screening programs for the detection of glaucoma. However, given the relatively low prevalence in the general population and the cost of the explorations performed, attempts at screening have remained isolated since they do not appear to be cost-effective [18]. But the progressive expansion of nonmydriatic fundus photography for diabetic retinopathy screening is providing the graders with high-quality photographs in which the optic nerve head can be readily assessed for glaucomatous damage, at no additional cost.

The idea of employing nonmydriatic fundus photography for glaucoma screening is not new. As early as 1990, a study was performed in which 183 first-degree relatives of glaucoma patients were photographed by a technician with a nonmydriatic fundus camera. The images were examined by an ophthalmologist for glaucomatous damage; 31 subjects (17%) were referred to further examinations, leading to the diagnosis of glaucoma in 6 cases (3%) [19]. Detry-Morel et al. evaluated the usefulness of nonmydriatic fundus photography, combined with frequency doubling perimetry and IOP measurement for detecting glaucoma in a general population. A total of 1620 subjects were included in the study; glaucomatous optic discs were detected in 3.5% of the subjects [20]. Steele et al. evaluated the optic nerve for signs of glaucomatous damage in nonmydriatic photographs taken for diabetic retinopathy screening; 1.42% were considered to have optic disc changes compatible with glaucoma [21]. Recently, the results of a study carried out to investigate the positive predictive value of the glaucoma referral process from a local unit of the English National Screening Programme for Diabetic Retinopathy (DRSP) have been reported. Of 11,565 diabetic patients screened, 216 were suspected to have glaucoma (1.87%). After independent grading by a glaucoma specialist, a total of 170 were graded glaucoma positive and referred to a clinic for further evaluation. After one year, 113 were found to be true cases and 22 were false cases. The authors concluded that optic discs imaging could be useful as part of a glaucoma screening strategy to identify the new disease within a diabetic population [10]. However, the first step towards evaluating the possible use of a diagnostic tool as a screening method is defining its accuracy for the diagnosis of the disease. The purpose of our study was to evaluate the accuracy of a set of criteria in evaluating the optic nerve head in nonmydriatic fundus photography for the diagnosis of glaucoma in diabetic patients (adapted from O'Connor et al. [13]). Although wedge defects of the RNFL are typically considered as glaucomatous damage signs, the RNFL defects are best detected with red-free and black-and-white fundus photograph [22]. For this reason only glaucomatous optic nerve signs of damage were considered in the present study.

A case-control study was chosen because, even in diabetics, the reported rate of glaucoma is low (5.5% [23]). When deciding on the criteria for glaucomatous optic nerve damage, the cut-off value for bilateral C/D ratio was chosen as 0.6 because when C/D ratio equals or exceeds 0.6, the probability of abnormality increases dramatically [24]. The side difference in C/D ratio was set at ≥0.2 because 88% of normal subjects have a C/D vertical ratio side difference equal or less than 0.1 [25]. The appearance of the optic nerve head was not used as a restriction criterion for the entry of subjects into either the normal or glaucoma groups. This tried to avoid sample bias that might influence the outcome.

In the present study, we have found that the assessment by two glaucoma specialists of nonmydriatic fundus photographs taken by trained nurses and forwarded telematically to a tertiary care hospital performs well for the diagnosis of glaucoma, with a sensitivity of 72.22%–79.17% and a specificity of 80.56%–88.88%. Overall accuracy was close to 80%. This compares favourably with previous reports of subjective assessment of stereophotographs [14–16, 26] and even with newer imaging devices [27–29]. In fact, in a high proportion of the images misclassified, the C/D ratio had been estimated correctly. These images corresponded to healthy eyes with macrodiscs or to glaucomatous eyes with no increased cupping.

Greaney et al. compared the ability of qualitative assessment by glaucoma specialists of optic nerve head stereophotographs, confocal scanning laser ophthalmoscopy (CSLO), scanning laser polarimetry (SLP), and OCT to distinguish normal eyes from those with early to moderate glaucomatous visual field defects. The sensitivity of stereophotograph grading ranged between 76 and 86% and the specificity between 85 and 92%. No single quantitative imaging technique, CSLO, SLP, or OCT, was better than qualitative assessment by a glaucoma specialist [28]. However, it must be taken into account that the accuracy of subjective assessment depends on the experience of the observer. Thus, Reus et al. performed a study in which 243 of 875 invited ophthalmologists in 11 European countries evaluated the stereoscopic slides of 40 healthy eyes and 48 glaucomatous eyes with varying severity and classified them as normal or glaucomatous. The overall accuracy was of 80.5%, with a sensitivity of 74.7% and a specificity of 87.4%. Imaging devices outperformed general ophthalmologists for the diagnosis of glaucoma; however, if glaucoma specialist assessments were considered, they were found to be slightly better than imaging devices [30]. In another study, it was found that glaucoma specialists classified the optic discs better than general ophthalmologists, who in turn outperformed hospital-based optometrists. The worst classifiers were junior residents [29]. Monoscopic digital images taken by a nonmydriatic fundus camera and forwarded telematically to a reading center have the advantage of being easier and quicker to acquire than stereoscopic images. We have shown that evaluation by a glaucoma specialist reaches a high accuracy for the diagnosis of glaucoma.

In summary, this is, as far as we could ascertain, the first study to evaluate the accuracy of monoscopic images taken with nonmydriatic fundus photography for the diagnosis of glaucoma in diabetic patients. The accuracy we have found is comparable to other imaging methods for glaucoma and may be high enough for it to be included in a screening program, in combination with other diagnostic techniques, although the best method for screening and whether screening is feasible requires further studies. An important setback of our study is that it is a case-control study with a very high proportion of patients compared with controls, when in a screening setting for glaucoma the prevalence of the disease would be much lower.

## Figures and Tables

**Table 1:** Distribution of signs of optic nerve damage in nonmydriatic fundus photography as evaluated by glaucoma specialist 1.

Signs of optic nerve damage		Controls (*n* = 72)	Glaucoma (*n* = 72)	Sensitivity and specificity
Vertical C/D ratio ≥0.6	No	65	24	Sensitivity 66.67% (55.78–77.56%)
	Yes	7	48	Specificity 90.28% (88.43–97.12%)

Vertical C/D ratio asymmetry ≥0.2	No	65	30	Sensitivity 58.33% (46.95–69.72%)
	Yes	7	42	Specificity 90.28% (83.43–97.12%)

Notches or thinning of the neuroretinal rim	No	72	32	Sensitivity 55.56% (44.08–67.03%)
	Yes	0	40	Specificity 100%

Optic disc hemorrhages	No	72	68	Sensitivity 5.56% (0.26–10.85%)
	Yes	0	4	Specificity 100%

C/D: cup to disc.

**Table 2:** Distribution of signs of optic nerve damage in nonmydriatic fundus photography as evaluated by glaucoma specialist 2.

Signs of optic nerve damage		Controls (*n* = 72)	Glaucoma (*n* = 72)	Sensitivity and specificity
Vertical C/D ratio ≥0.6	No	67	39	Sensitivity 45.83% (34.32–57.34%)
	Yes	7	33	Specificity 93.05% (87.18–98.92%)

Vertical C/D ratio asymmetry ≥0.2	No	66	37	Sensitivity 48.61% (37.06–60.15%)
	Yes	6	35	Specificity 91.66% (85.28–98.05%)

Notches or thinning of the neuroretinal rim	No	70	48	Sensitivity 33.33% (24.44–44.02%)
	Yes	2	24	Specificity 97.22% (93.42–101.01%)

Optic disc hemorrhages	No	72	68	Sensitivity 5.56% (0.26–10.85%)
	Yes	0	4	Specificity 100%

C/D: cup-to-disc.
